# Advanced Glycation in macrophages induces intracellular accumulation of 7-ketocholesterol and total sterols by decreasing the expression of ABCA-1 and ABCG-1

**DOI:** 10.1186/1476-511X-10-172

**Published:** 2011-09-29

**Authors:** Rodrigo T Iborra, Adriana Machado-Lima, Gabriela Castilho, Valeria S Nunes, Dulcinéia SP Abdalla, Edna R Nakandakare, Marisa Passarelli

**Affiliations:** 1 Lipids Laboratory (LIM-10), Faculty of Medical Sciences, University of São Paulo. São Paulo, Brazil; 2 Department of Clinical and Toxicology Analysis, Faculty of Pharmaceutical Sciences, University of São Paulo, São Paulo, Brazil

**Keywords:** advanced glycation, diabetes mellitus, oxysterols, 7-ketocholesterol; ABCA-1, ABCG-1

## Abstract

**Background:**

Advanced glycation end products (AGE) alter lipid metabolism and reduce the macrophage expression of ABCA-1 and ABCG-1 which impairs the reverse cholesterol transport, a system that drives cholesterol from arterial wall macrophages to the liver, allowing its excretion into the bile and feces. Oxysterols favors lipid homeostasis in macrophages and drive the reverse cholesterol transport, although the accumulation of 7-ketocholesterol, 7alpha- hydroxycholesterol and 7beta- hydroxycholesterol is related to atherogenesis and cell death. We evaluated the effect of glycolaldehyde treatment (GAD; oxoaldehyde that induces a fast formation of intracellular AGE) in macrophages overloaded with oxidized LDL and incubated with HDL alone or HDL plus LXR agonist (T0901317) in: 1) the intracellular content of oxysterols and total sterols and 2) the contents of ABCA-1 and ABCG-1.

**Methods:**

Total cholesterol and oxysterol subspecies were determined by gas chromatography/mass spectrometry and HDL receptors content by immunoblot.

**Results:**

In control macrophages (C), incubation with HDL or HDL + T0901317 reduced the intracellular content of total sterols (total cholesterol + oxysterols), cholesterol and 7-ketocholesterol, which was not observed in GAD macrophages. In all experimental conditions no changes were found in the intracellular content of other oxysterol subspecies comparing C and GAD macrophages. GAD macrophages presented a 45% reduction in ABCA-1 protein level as compared to C cells, even after the addition of HDL or HDL + T0901317. The content of ABCG-1 was 36.6% reduced in GAD macrophages in the presence of HDL as compared to C macrophages.

**Conclusion:**

In macrophages overloaded with oxidized LDL, glycolaldehyde treatment reduces the HDL-mediated cholesterol and 7-ketocholesterol efflux which is ascribed to the reduction in ABCA-1 and ABCG-1 protein level. This may contribute to atherosclerosis in diabetes mellitus.

## Background

Oxysterols are oxidized derivatives of cholesterol that act as important mediators of lipid metabolism, particularly by driving intracellular lipid homeostasis and the reverse cholesterol transport [1,2]. Nonetheless, the accumulation of 7-ketocholesterol, 7alpha-hydroxycholesterol and 7beta-hydroxycholesterol has been implicated in the development of atherosclerosis as wells as inflammation and macrophage foam cell death [3].

In diabetes mellitus, advanced glycation end products (AGE) impair lipid metabolism and reverse cholesterol transport by diminishing the expression of ABCA-1, ABCG-1 and the activity of lecithin cholesterol acyltransferase [4-6]. On the hand, glycation enhances cholesteryl ester transfer protein activity making more cholesterol available for arterial wall macrophage by the uptake of modified LDL by scavenger receptors [7]. There are no data available on the role played by intracellular advanced glycation elicited by the treatment with oxoaldehydes - that induce a rapid formation of AGE - in the intracellular content of oxysterols subspecies. Then the aim of this study was to analyze in oxidized LDL-overloaded macrophages treated with HDL alone or HDL plus LXR agonist (T0901317) the effect of advanced glycation in the selective distribution of oxysterols and the total content of sterols as well as the expression of ABCA-1 and ABGC-1.

## Methods

### Lipoprotein isolation and oxidative modification

Low density lipoprotein (LDL; d = 1.019-1.063 g/mL) and high density lipoprotein (HDL; d = 1.063-1.121 g/mL) were isolated after sequential preparative ultracentrifugation of fresh plasma drawn from healthy donors and were purified by discontinuous gradient ultracentrifugation. After dialysis, all lipoproteins fractions were sterilized in a 0.22 μm filter. LDL was oxidized according to Steinbrecher et al. [8] and protein was measured by the Lowry technique [9]

### Cell Culture and Experimental Protocol

J774 macrophages were cultured in RPMI 1640 medium (Gibco, Grand Island, New York, USA) containing 10% fetal calf serum (Cultilab, Campinas, Brazil), penicillin and streptomycin (Gibco), and maintained in a 5% CO2 incubator at 37°C. After reaching confluence, macrophages were enriched with oxidized LDL (50 μg/mL of low glucose DMEM - Gibco) for 48h and then treated with 0.5 mM glycolaldehyde (Sigma Chem. Com. St. Louis, USA) (GAD-macrophages) or DMEM alone (C-macrophages) for the last 5h, in the absence or presence of the LXR-agonist, T0901317 (1 μM). Cells were carefully washed and incubated with 50 μg/mL of HDL (5 h) in the absence or presence of T0901317.

### Oxysterols and total cholesterol determination

Sterol standards (cholesterol, 7alpha-hydroxycholesterol, 7beta-hydroxycholesterol, 7-ketocholesterol, colestan-3beta, 5alpha, 6beta-triol, 5alpha, 6alpha-epoxycholesterol, 5beta, 6beta-epoxycholesterol, 24(s), 25 epoxycholesterol, 25-hydroxycholesterol and 27-hydroxycholesterol), including internal standard 5beta-cholestane, were obtained from Steraloids Inc. (Wilton, NH, USA).

Cellular lipids were extracted with hexane:isopropanol (3:2, v/v). To the lipid extract it was added 10 μg of 5alpha-cholestane (internal standard) following evaporation under nitrogen. Samples were redissolved in 1.0 mL toluene/ethyl acetate (1 : 1, v/v) and applied to diol extraction columns (Waters Corporation, Ireland), previously conditioned with the same solvent. After collection of the first eluent fraction under mild vacuum, columns were eluted with 2.0 mL of toluene/ethyl acetate and the eluent was pooled with the original fraction. The toluene/ethyl acetate fraction was evaporated to dryness under nitrogen, and redissolved in 2.0 mL diethyl ether and subjected to a two-phase alkaline saponification procedure. The recovered ether phase was dryness under nitrogen and derivatized after additions of 100 μL pyridine (Merck, Rio de Janeiro, Brazil) and *N*,*O*-bis(trimethylsylil) trifluoroacetamide (BSTFA; Supelco - Bellefonte, USA). Vials were sealed, purged with nitrogen, and heated to 60°C for 60 min [10]. Samples were transferred to tubes with ether, followed by gas chromatography-Mass Spectrometer detection (GC-MS) analysis. One μL aliquot was then injected into a QP 2010 plus (Shimadzu - Kyoto, Japan) gas chromatograph equipped with Rxi-1ms de 30 m (Restec - Bellefonte, EUA) and Mass Spectrometer detector. Helium was used as the carrier gas at a flow rate of 5 mL/min; the injection temperature was set at 290°C and the initial column temperature at 240°C, with a split ratio of 1:5. The ion detector temperature was set at 300°C and a programmed temperature run was used with the initial temperature held for 1 min followed by a 5°C/min temperature ramp to 290°C, with the final temperature held for 20 min [10].

### Immunoblot

J774 Macrophages were scraped into Tris buffer saline containing protease inhibitors. Equal amounts of cell protein were applied to a 6% polyacrylamide gel (SDS-PAGE) and blotted to Amersham Hybond ECL Nitrocellulose Membrane (GE healthcare, Little Chalfont, UK). The blots were treated with defatted dry milk (5%) in PBS/0.05% Tween 20, and incubated for 3h with primary antibodies, anti ABCA-1 or anti ABCG-1, 1:1000 (Novus Biologicals, Inc., Littleton, USA) in blocking buffer. After washing three times with TBS/0.05% Tween 20, membranes were incubated with peroxidase-conjugated anti-mouse or anti-rabbit antibodies (GE healthcare) for 2 h, washed and developed with a SuperSignal West Pico Chemiluminescent Substrate (Termo Scientific Rockford, IL, EUA). Chemiluminescence was detected by ImageQuant 350 (GE Healthcare, Piscata Way, NJ, USA). Data are presented as arbitrary units corrected per beta-actin 1: 1000 (Fitzgerald industries int., Acton, MA, USA).

### Statistical analysis

Statistical analyses were performed using GraphPad Prism 4.0 software (GraphPad Prism, Inc., San Diego, CA). Student's T test was utilized to compare results (mean ± SD). One way ANOVA and Bonferroni post test (mean ± SD) was utilized to compare results among groups. A p-value < 0.05 was considered statistically significant.

## Results

The content of oxysterols and total cholesterol in oxidized LDL and native HDL utilized in cell incubations is shown in Table 1. In oxidized LDL the amount of oxysterols was superior to cholesterol as expected by the chemical modification induced in vitro. Also, the higher amount of oxysterols was represented by 7-ketocholesterol (40.9%), 7beta-hydroxycholesterol (21.3%), 7alpha-hydroxycholesterol (20.5%) and cholesterol-5,6beta (9.1%). In HDL we only observed a very small amount of oxysterols.

**Table 1:** Oxysterols and total cholesterol in oxidized LDL and native HDL (ng/μg of protein ± DP)

	LDLox	HDL
	(n = 5)	(n = 2)
**7-ketocholesterol**	**73.1 ± 18**	**0.96 ± 0.70**
**7β-hydroxycolesterol**	**38.1 ± 8**	**0.64 ± 0.13**
**7α-hydroxycolesterol**	**36.6 ± 8**	**0.38 ± 0.11**
**cholesterol- 5,6 α**	**16.16 ± 4**	**0.17 ± 0.04**
**cholesterol-5,6 β**	**12.97 ± 11**	**0.01 ± 0.02**
**cholestan-3β,5α,6β-triol**	**1.321 ± 0.3**	**0.19 ± 0.01**
**24.25 epoxycholesterol**	**ND**	**1.90 ± 0.22**
**25-hydroxycholesterol**	**1.06 ± 0.2**	**0.03 ± 0.01**
**27-hydroxycholesterol**	**0.11 ± 0.07**	**0.02 ± 0.01**
**Total oxysterols**	**179.4 ± 39**	**5.83 ± 2.79**
**Total cholesterol**	**99.1 ± 7**	**93.73 ± 0.89**

**Figure 1:**
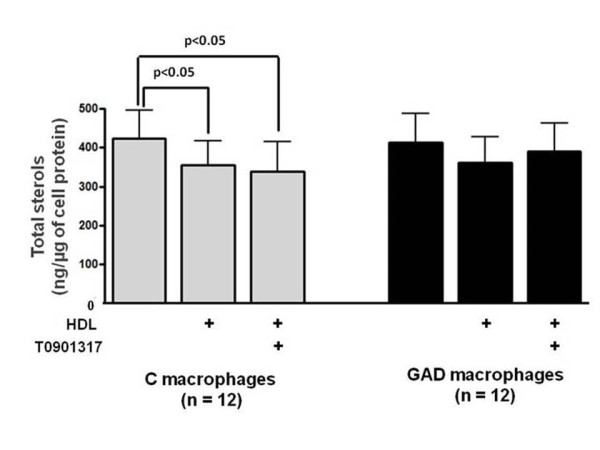
**Total sterols content in control and glycolaldehyde-treated macrophages**. J774 macrophages were overloaded with oxidized LDL (50 μg/mL de DMEM) for 48 h. In the last 5h, cells were incubated with 0.25 mM glycolaldehyde (GAD; black bars) and 1 μM LXR agonist (T0901317). Control cells (C; gray bars) were incubated with T0901317 alone. After washing, macrophages were incubated for 5 h with HDL alone (50 μg/mL de DMEM) or HDL + T0901317. Total sterols were determined after lipid cell extraction by CG/MS, utilizing 5-alpha cholestane as an internal standard.

In control macrophages the presence of HDL or HDL + T0901317 reduced, respectively, 16% and 22% the amount of intracellular total sterols (oxysterols + cholesterol) in comparison to cells maintained in the presence of culture medium alone. No differences were found in the amount of total sterols when comparing control macrophages treated with HDL and HDL + T0901317 (Figure 1). In opposition, in GAD macrophages the addition of HDL or HDL + T0901317 was not able to reduce intracellular content of total sterols (Figure 1 ).

**Figure 2:**
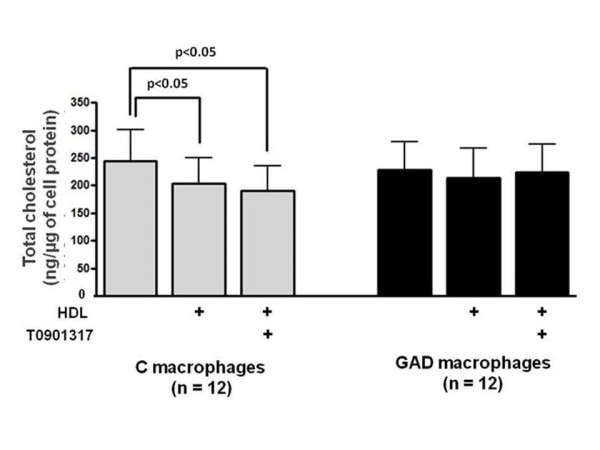
**Total cholesterol content in control and glycolaldehyde-treated macrophages**. J774 macrophages were overloaded with oxidized LDL (50 μg/mL de DMEM) for 48 h. In the last 5h, cells were incubated with 0.25 mM glycolaldehyde (GAD; black bars) and 1 μM LXR agonist (T0901317). Control cells (C; gray bars) were incubated with T0901317 alone. After washing, macrophages were incubated for 5 h with HDL alone (50 μg/mL de DMEM) or HDL + T0901317. Total cholesterol was determined after lipid cell extraction by CG/MS, utilizing 5-alpha cholestane as an internal standard.

**Figure 3:**
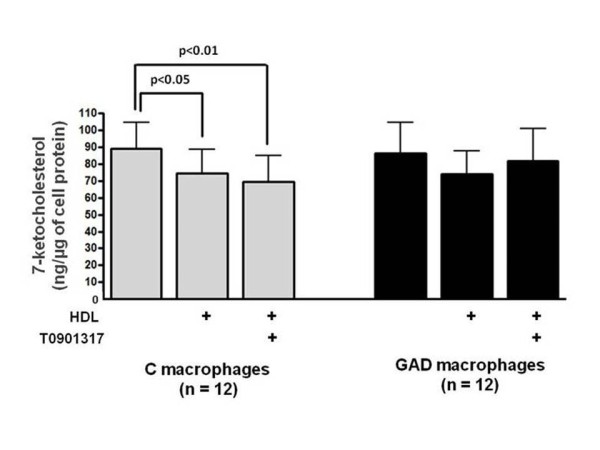
**7-ketocholesterol content in control and glycolaldehyde-treated macrophages**. J774 macrophages were overloaded with oxidized LDL (50 μg/mL de DMEM) for 48 h. In the last 5h, cells were incubated with 0.25 mM glycolaldehyde (GAD; black bars) and 1 μM LXR agonist (T0901317). Control cells (C; gray bars) were incubated with T0901317 alone. After washing, macrophages were incubated for 5 h with HDL alone (50 μg/mL de DMEM) or HDL + T0901317. 7-ketocholesterol was determined after lipid cell extraction by CG/MS, utilizing 5-alpha cholestane as an internal standard.

Intracellular cholesterol content was also reduced in control macrophages after incubation with HDL (17%) or HDL + T0901317 (22%) which was not observed in GAD-macrophages (Figure 2). In addition, in control macrophages, HDL and HDL + T0901317, respectively diminished, 16% e 21% the intracellular content of 7-ketocholesterol, without affecting other oxysterols. On the other hand, 7-ketocholesterol was not reduced in GAD-macrophages after incubation with HDL or HDL + T0901317 (Figure 3 and Table 2).

**Table 2:** Oxysterols and total cholesterol in control (C) and glycolaldehyde-treated macrophages (GAD)

Macrophage		C			GAD	
	-	**HDL**	**HDL** **+** **T0901317**	-	**HDL**	**HDL** **+** **T0901317**

**7-ketocholesterol**	**86.7 ± 16**	72.8 ± 14*	68.4 ± 14*	**83.8 ± 19**	**74.2 ± 13**	**79.8 ± 19**
**7β-hydroxicholesterol**	**26.7 ± 9**	**20.4 ± 6**	**21.5 ± 10**	**25.5 ± 10**	**23.6 ± 6**	**22.8 ± 10**
**7α-hydroxicholesterol**	**9.2 ± 4**	**8.1 ± 3**	**8.1 ± 5**	**9.4 ± 3**	**8.8 ± 2**	**8.6 ± 4**
**cholesterol-5,6 β**	**22.6 ± 9**	**20 ± 5**	**16.9 ± 6**	**24.2 ± 6**	**22.9 ± 6**	**20.5 ± 6**
**cholesterol- 5,6 α**	**7.1 ± 2**	**6.6 ± 2**	**5.5 ± 2**	**7.4 ± 2**	**6.2 ± 2**	**6.7 ± 3**
**24.25 epoxycholesterol**	**3.8 ± 2**	**3.5 ± 2**	**2.8 ± 1**	**3.9 ± 2**	**3.7 ± 2**	**3.5 ± 2**
**27-hydroxycholesterol**	**0.48 ± 0.2**	**0.51 ± 0.3**	**0.44 ± 0.2**	**0.55 ± 0.3**	**0.52 ± 0.2**	**0.48 ± 0.2**
**25-hydroxycholesterol**	**0.35 ± 0.3**	**0.38 ± 0.1**	**0.42 ± 0.2**	**0.43 ± 0.2**	**0.37 ± 0.1**	**0.40 ± 0.2**
**cholestan-3β,5α,6β-triol**	**10 ± 3**	**8.3 ± 2**	**8.5 ± 5**	**10.5 ± 4**	**9.4 ± 3**	**9.2 ± 3**
**total cholesterol**	**244 ± 57**	203 ± 47*	191 ± 44*	**227 ± 52**	**214 ± 53**	**224 ± 50**

Values were corrected by 5-alpha cholestane (internal standard) and are expressed as ng/μg of cell protein ± DP (n = 12).

* p < 0.05 comparison were made between cells maintained in the presence of culture medium alone with HDL or HDL + T0901317. One way ANOVA and Bonferroni post test (mean ± SD).

**Figure 4:**
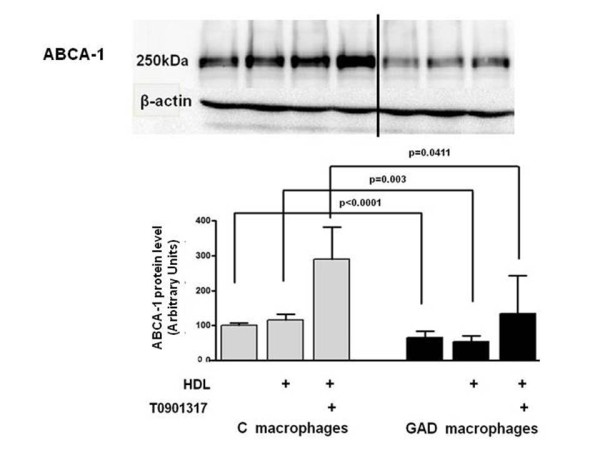
**ABCA-1 protein level in control and glycolaldehyde-treated macrophages**. J774 macrophages were overloaded with oxidized LDL (50 μg/mL de DMEM) for 48 h. In the last 5h, cells were incubated with 0.25 mM glycolaldehyde (GAD; black bars) and 1 μM LXR agonist (T0901317). Control cells (C; gray bars) were incubated with T0901317 alone. Cells were scrapped into TBS containing protease inhibitors. Equal amounts of cell protein were applied into a 6% polyacrylamide electrophoresis gel and immunoblot was performed utilizing anti ABCA-1 antibody. Data (arbitrary units; n = 4) were corrected per beta-actin.

In oxidized LDL overloaded control and GAD-macrophages the ABCA-1 protein level was, respectively, 2.8 and 1.8 times enhanced in comparison to non-overloaded cells (data not shown). In the presence of HDL or HDL + T0901317, control macrophages presented a 2.9 and 2.4 fold enhancement in ABCA-1 content, in comparison to cells maintained in culture medium alone (Figure 4). In comparison to C, GAD-macrophages presented a smaller amount of ABCA-1 (45%) with no increment in protein level attained after incubation with HDL or HDL + T0901317 (Figure 4).

**Figure 5:**
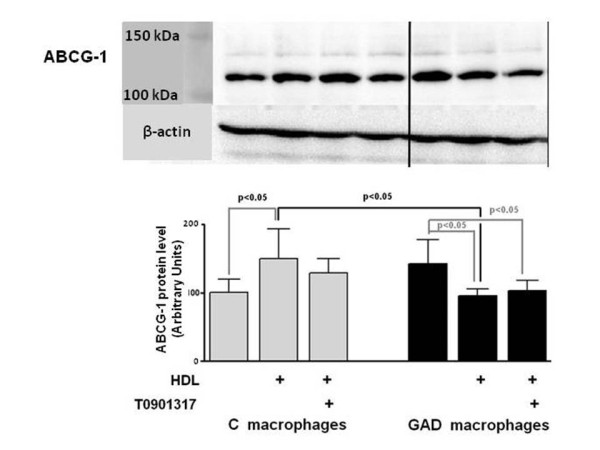
**ABCG-1 protein level in control and glycolaldehyde-treated macrophages**. J774 macrophages were overloaded with oxidized LDL (50 μg/mL de DMEM) for 48 h. In the last 5h, cells were incubated with 0.25 mM glycolaldehyde (GAD; black bars) and 1 μM LXR agonist (T0901317). Control cells (C; gray bars) were incubated with T0901317 alone. Cells were scrapped into TBS containing protease inhibitors. Equal amounts of cell protein were applied into a 6% polyacrylamide electrophoresis gel and immunoblot was performed utilizing anti ABCG-1 antibody. Data (arbitrary units; n = 4) were corrected per beta-actin.

ABCG-1 level was 30% reduced in control macrophages treated with oxidized LDL in comparison to non-overloaded cells. In control macrophages previously enriched with oxidized LDL, the presence of HDL was able to enhance ABCG-1 (49.7%), without further changes by the addition of T0901317 (Figure 5).

As compared to oxidized LDL non-overloaded cells, in GAD-macrophages, oxidized LDL enrichment did not change cell content of ABCG-1. In this case, the presence of HDL diminished 36.6% the amount of ABCG-1 in comparison to control cells treated with this lipoprotein. Also, in GAD-macrophages the incubation with HDL and HDL + T0901317 reduced the protein level of ABCG-1 as compared to GAD-cells maintained in culture medium alone (Figure 5**)**.

## Discussion

Advanced glycation end products are independently associated with the development of atherosclerosis [11]. Oxysterols, mainly 24(s),25 epoxycholesterol, 22(R)-hydroxycholesterol, o 24(S)-hydroxycholesterol and 25 hydroxycholesterol [12], modulate intracellular cholesterol exportation by increasing the LXR-mediated expression of ABCA-1 and ABCG-1 [13]. On the other hand, *7*-oxygenated cholesterol derivatives such as 7-ketocholesterol, are related to cell toxicity and death by inducing inflammation and endoplasmic reticulum stress [14]. In fact, these oxysterols are prevalent in oxidized LDL and its accumulation in arterial wall macrophages is a hallmark of atherosclerosis and plaque instability [15,16].

Oxidized LDL utilized to overload macrophages with lipids previously to the incubation with HDL and LXR agonist was in fact enriched in 7-ketocholesterol [17] due to the no enzymatic conversion of cholesterol into oxysterols. HDL on its turn presented very small amounts of oxysterols maybe related to the acquisition of these components from other lipoproteins before plasma ultracentrifugation [18].

In cells grown in the absence of oxidized LDL only a very small amount of oxysterols was detected by CG/MS in regard to the total amount of cell cholesterol (data not shown). Then, the majority of oxysterols that we found in oxidized LDL-overloaded macrophages was originated from the uptake of the modified LDL.

We found that advanced glycation in macrophages induced by the treatment with glycolaldehyde disturbs the exportation of 7-ketocholesterol, inducing its intracellular accumulation. Interestingly, GAD treatment mitigates the HDL ability to remove cell cholesterol and 7-ketocholesterol even in the presence of T0901317. In cholesterol-enriched macrophages, ABCA-1 contributes to a large amount of cholesterol net efflux to lipid-poor apolipoprotein A-I generating pre- betaHDL. In this present study a reduced amount of cellular ABCA-1 protein level was observed in GAD-macrophages with a small increment in its expression even after LXR agonist treatment.

We have recently demonstrated that advanced glycated albumin isolated from poorly controlled diabetes mellitus' serum reduces protein content of ABCA-1 and ABCG-1 in macrophages, impairing cholesterol efflux and inducing intracellular lipid accumulation [19]. It has been previously shown that advanced glycation reduces ABCG-1 mRNA levels by impairing *ABCG-1 *gene transcriptional activity independently of LXR [6]. On the other hand, advanced glycation induced both by oxoaldehyde or advanced glycated albumin treatment reduces ABCA-1 protein levels in macrophages which are not related to alterations in ABCA-1 mRNA levels [6,20]. The mechanisms that regulate ABCA-1 final protein content in cells submitted to advanced glycation are not completely understood so far but may involve post-translational modifications of ABCA-1. Previous data from our group showed that advanced glycated albumin induces the expression of endoplasmic reticulum stress and unfolded protein response markers in macrophages. Interestingly, a chemical chaperone (4-phenylbutyric acid) that alleviates endoplasmic reticulum stress is able to recover ABCA-1 level and apo A-I- mediated cholesterol efflux in these cells [14]. These events were also related to the elevated generation of reactive oxygen species in glycolaldehyde-modified albumin-treated macrophages, since treatment with aminoguanidine that reduced ROS generation was able to restore ABCA-1 protein content [21]. AGE-albumin also primers macrophages to inflammation which is related to the diminished cholesterol exportation to apolipoprotein A-I [22]. Although we were not able to detect by immunoblot CML derivatives in ABCA-1 and ABCG-1, at this point we cannot completely exclude that part of the changes observed in the content and activity of ABCA-1 and ABCG-1 relates to GAD modification of both transporters.

Oxidative stress and inflammation induced by AGE trigger endoplasmic reticulum stress that is also observed in instable atherosclerotic plaques enriched in 7-ketocholesterol. More recently, Yehuda et al [23] have demonstrated that a lipid extract obtained from carotid atherosclerotic lesion induces the expression of inflammatory markers in macrophages, which was mainly attributed to the cholesterol and oxysterol-enriched fraction (7alpha-hydroxycholesterol, 7beta-hydroxycholesterol, 7-ketocholesterol and 26-hydroxycholesterol) of the lipid extract.

ABCG-1 plays a major role in the efflux of 7-ketocholesterol to large HDL minimizing its apoptotic effects in macrophages. Nonetheless, in ABCG-1 knockout mice the development of atherosclerosis is still controversial. It is possible that in those animal models the compensatory expression of ABCA-1 may abrogates the absence of ABCG-1-mediated lipid efflux. In the setting of hyperglycemia, AGE formation is prevalent compromising both the expressions of ABCA-1 and ABCG-1. Then, the accumulation of 7-ketocholesterol together with total sterols may aggravate the development of atherosclerosis in diabetes mellitus.

## Competing interests

The authors declare that they have no competing interests.

## Authors' contributions

RTI carried out lipoprotein isolation and oxidative modification, cell culture, oxysterols and total cholesterol determination, protein quantification, statistical analysis and participated in preparation of the manuscript. AML carried out glycation proceeds and participated in cell culture. GC participated in protein quantification. VSN participated in oxysterols and total cholesterol determination

DSPA provide oxysterols standards and protocols. ERN participated in experimental design and statistical analysis. MP was responsible for experimental design, coordination of research and preparation of the manuscript.
